# Concentrated growth factor combined with iRoot BP Plus promotes inflamed pulp repair: an in vitro and in vivo study

**DOI:** 10.1186/s12903-023-02903-5

**Published:** 2023-04-19

**Authors:** Qian Zeng, Can Zhou, Mengjie Li, Yu Qiu, Xi Wei, Hongyan Liu

**Affiliations:** 1 Hospital of Stomatology, Sun Yat-Sen University; Guangdong Provincial Key Laboratory of Stomatology, Guangzhou, 510055 China; 2 Guanghua School of Stomatology, Sun Yat-Sen University, Guangzhou, 510055 China

**Keywords:** Pulpitis, Vital pulp therapy, Concentrated growth factor, Bioactive calcium silicate cements

## Abstract

**Background:**

Platelet concentrates combined with calcium silicate cements may promote reparative dentin formation. However, few studies have reported their effect on dental pulp inflammation. This study aimed to evaluate the effects of concentrated growth factor (CGF) combined with iRoot BP Plus on inflammatory human dental pulp stem cells (hDPSCs) in vitro and inflamed pulp in rats in vivo.

**Methods:**

The proliferation of LPS-stimulated hDPSCs treated with 50% CGF with/without 25% iRoot BP Plus was evaluated using Cell Counting Kit-8 on days 1, 4 and 7. The expression of genes associated with inflammation on day 1 and differentiation on day 14 was analysed by real-time polymerase chain reaction. The exposed pulp of rat maxillary molars was injected with 10 mg/mL LPS and directly capped with CGF membrane with/without iRoot BP Plus extract for 1, 7 and 28 days. The teeth were subjected to histologic analyses and immunohistochemistry.

**Results:**

The proliferation rates of the inflammatory hDPSCs after the combination treatment were significantly higher than those after the other treatments on days 4 and 7 (*P* < 0.05). IL-1β, IL-6, and TNF-α levels were increased in inflammatory hDPSCs but decreased after treatment with CGF combined with iRoot BP Plus extract, whereas IL-4 and IL-10 showed the opposite expression patterns. Expression of the odontogenesis-related genes OCN, Runx2, and ALP was dramatically enhanced by combined treatment with CGF and iRoot BP Plus extract. In rat pulp, the average inflammation scores of the CGF and CGF-iRoot BP Plus groups significantly decreased in comparison with those of the LPS group (*P* < 0.05), and the CGF-iRoot BP Plus group had more reparative dentin than the CGF and BP groups. Immunohistochemical staining showed fewer M1 macrophages on day 1 and more M2 macrophages on day 7 in the CGF-iRoot BP Plus group than in the other groups.

**Conclusions:**

The combination of CGF and iRoot BP Plus showed a synergistic effect on anti-inflammatory potential and promoted greater pulp healing than CGF or iRoot BP Plus alone.

## Background

In recent years, vital pulp therapy (VPT) has been reinvigorated due to the advent of bioactive calcium silicate cements and improvements in the understanding of pulpal immunity and biology. The position statements of the European Society of Endodontology (ESE) and American Association of Endodontists (AAE) have suggested that VPT may be used in mature teeth even with symptomatic pulp [1, 2]. Several studies have also verified the efficacy of VPT for mature teeth with irreversible pulpitis caused by caries [3, 4]. In these cases, precise removal of infected pulp tissue and preservation of healthy pulp are the critical steps of VPT. However, this procedure can be a challenge for practitioners, as the depth of infected pulp tissues is difficult to assess [5]. Earlier findings and our previous study have shown that biomarkers for pulp inflammation may help identify specific pathological conditions associated with pulpitis [6, 7]. However, direct correlations between biomarkers and histologic diagnoses have not been established. Recently, the question of how to control pulp inflammation and promote pulp tissue repair using pulp capping materials has attracted substantial interest in this field [8].

Ideal pulp capping materials should have excellent biocompatibility, good sealing ability, ease of handling, and the ability to stimulate dentin bridge formation [9]. Calcium silicate cements, including mineral trioxide aggregate (MTA), iRoot BP Plus, and Biodentine, have been used as pulp capping materials in VPT [10, 11]. For carious exposed pulp diagnosed with reversible pulpitis, calcium silicate cements were reported to have a high success rate with direct pulp capping (DPC) [12]. Nevertheless, pulpotomy with calcium silicate cements has been suggested for symptomatic irreversible pulpitis, which involves removal of infected and irreversibly inflamed pulp tissue [2]. In comparison with pulpotomy, DPC preserves the structural integrity and immunological functions of the tooth, thus resulting in more predictable healing and pulp sensitivity responses after treatment [13]. We hypothesized that if pulp inflammation can be reversed or arrested in cases of irreversible pulpitis, DPC may be advantageous. Recently, newly developed and refined biologically based wound-healing strategies have been proposed to improve the outcomes of VPT [8]. The optimization of pulp capping agents is imperative for VPT outcomes. Therefore, successful DPC treatment under unpredictable inflammatory conditions in pulp tissue requires pulp capping materials with greater efficacy to modulate the inflammatory response and reestablish normal pulp.

Concentrated growth factor (CGF), the latest generation of platelet concentrate products, has been used as a bioscaffold for tissue regeneration [14–17]. CGF has a relatively stiff structure similar to that of natural fibrin endowed with white blood cells, red blood cells and blood platelets [18, 19]. It contains abundant growth factors, including platelet-derived growth factor-BB (PDGF-BB), transforming growth factor β-1 (TGF-β1), insulin-like growth factor-1 (IGF-1), vascular endothelial growth factor (VEGF), and basic fibroblast growth factor (bFGF) [20, 21], which are involved in the regulation of cell migration, proliferation, differentiation, and angiogenesis and vital for tissue regeneration. Xu et al. found that CGF could reduce the expression of IL-8 and TNF-α in hDPSCs in vitro [22], indicating that CGF may regulate the inflammatory response of dental pulp. However, CGF has certain solubility and poor stability, which is not sufficient to continuously support tissue repair and regeneration [23]. In recent years, some studies have applied platelet concentrates combined with bioceramic materials as pulp capping materials [24, 25]. An in vitro study showed that compared with MTA or platelet-rich fibrin (PRF) alone, the composite pulp capping material of PRF combined with MTA could promote the expression of dentin sialophosphoprotein (DSPP) and dentin matrix protein-1 (DMP-1), enhance alkaline phosphatase (ALP) activity, and induce mineralization of human dental pulp cells [24]. When used as a DPC agent for rat molars with mechanical pulp exposure, the combination of PRF and MTA promoted the formation of reparative dentin [25], suggesting that this kind of new composite material that combines platelet concentrates with bioactive calcium silicate cements has potential as a pulp capping material. However, there are few reports about the effects of this new type of composite pulp capping material on the inflammatory response in dental pulp in an inflamed microenvironment.

The present study aimed to evaluate the effects of CGF combined with iRoot BP Plus on the inflammatory response and odontogenic differentiation ability of human dental pulp stem cells (hDPSCs) under inflamed conditions in vitro and the potential role of this composite biomaterial in inflamed rat dental pulp in vivo.

## Methods

The present study was approved by the Ethics Committee of the Affiliated Hospital of Stomatology, Sun Yat-sen University (protocol code: KQEC-2022–32-01) and the Institutional Animal Care and Use Committee of Sun Yat-Sen University (protocol code: SYSU-IACUC-2022–000,691).

### Isolation, culture and characterization of human dental pulp stem cells

Healthy impacted third molars were obtained from patients between 16 and 25 years old with informed consent at the Hospital of Stomatology, Sun Yat-sen University. DPSCs were isolated by enzyme digestion according to a previously described method [26]. The separated cells were cultured in α-minimum essential medium (αMEM; Gibco, USA) supplemented with 10% foetal bovine serum (FBS; Gibco BRL, USA), 100 U/ml penicillin-G (Sigma‒Aldrich, USA) and streptomycin in a humidified atmosphere of 5% CO_2_ at 37 °C. The cells started to grow out within 2 weeks. After the cells reached 90% confluence, they were dissociated and regarded as primary cells. Cells at passages 3–5 (P3-5) were used in this study.

Flow cytometry was performed to identify DPSCs. In brief, typical positive and negative surface markers of MSCs, including CD29-FITC, CD105-PE, CD90-PE, CD34-FITC and CD45-PE (BD Bioscience, USA), were evaluated. The isotype served as the negative control. Third-passage DPSCs were suspended at 5 × 10^5^ cells/mL in phosphate-buffered saline (PBS, Sigma‒Aldrich, USA), stained with different antibodies for 30 min at 4 °C, washed with PBS, resuspended in FACS buffer, and analysed using a MOFlo™ high-performance cell sorter (Beckman Coulter, USA). For the multilineage differentiation assay, alizarin red S staining and oil red O staining were used to identify mineralized nodules and lipid droplets after the cells were incubated with differentiation induction medium for 3 weeks.

### Conditioned medium preparation

Venous blood (10 mL) was collected from each participant who was a nonsmoker and in good general health after informed consent was obtained. Tubes of blood were processed in a Medifuge centrifuge device (Silfradent Srl, Sofia, Italy) to obtain CGF, following the manufacturer’s instructions: 30 s acceleration, 2 min at 2700 rpm (600 g), 4 min at 2400 rpm (400 g), 4 min at 2700 rpm (600 g), 3 min at 3000 rpm, and 36 s deceleration. After centrifugation, 3 layers were observed in the blood: the upper platelet-poor plasma layer, the middle fibrin-rich gel with aggregated platelets and concentrated growth factors, and the lower red blood cell layer. Then, the isolated CGF membrane was cut into small pieces and placed in a − 80 °C freezer for 1 h. After thawing at 4 °C and centrifugation for 10 min at 230 g, the exudates were harvested and immersed in 5 mL αMEM, which was defined as 100% CGF. Following incubation at 37 °C for 24 h and centrifugation for 5 min at 400 g, the supernatant was frozen at − 80 °C [27]. The 100% CGF was diluted with αMEM to produce 50% CGF for use in the in vitro study.

As previously reported [28], iRoot BP Plus (Innovative Bioceramix, Vancouver, Canada) was stored in a 100% humidified atmosphere at 37 °C for 3 days. After solidification, the materials were ground into a powder. Then, 1.0 g powder was dissolved in 50 mL αMEM for 1 day and centrifuged for 5 min at 3000 g. The obtained medium was defined as 100% iRoot BP Plus extract, and 25% iRoot BP Plus extract was obtained by dilution with αMEM. The medium was stored at 4 °C and applied in the following study.

### LPS treatment

The hDPSCs were seeded in a 12-well plate (Corning, USA) at a density of 1 × 10^5^ cells per well and treated with 0.1, 1 and 10 μg/mL LPS (Escherichia coli 0111:B4, Sigma) for 24 h when the cells grew to 80% confluence. Only culture medium was used as a negative control. Cell Counting Kit-8 (Dojindo, Japan) assays and real-time quantitative polymerase chain reaction (PCR) were performed to analyse cell proliferation and the expression of inflammation-related genes (IL-6, IL-1β and TNF-α). The primer sequences for IL-6, IL-1β, and TNF-α (BGI, Shenzhen, China) are listed in Table 1. After comprehensive analysis of the results, we selected 1 μg/mL LPS for the assays with inflamed hDPSCs.

**Table 1:** Primers used for real-time quantitative PCR

Gene	Forwards primer	Reverse primer
IL-4	CCAACTGCTTCCCCCTCTG	TCTGTTACGGTCAACTCGGTG
IL-6	ACTCACCTCTTCAGAACGAATTG	CCATCTTTGGAAGGTTCAGGTTG
IL-10	GACTTTAAGGGTTACCTGGGTTG	TCACATGCGCCTTGATGTCTG
IL-1β	ATGATGGCTTATTACAGTGGCAA	GTCGGAGATTCGTAGCTGGA
TNF-α	CGTGGAGCTGGCCGAGGAG	AGGAAGGAGAAGAGGCTGAGGAAC
OCN	AGCAAAGGTGCAGCCTTTGT	GCGCCTGGGTCTCTTCACT
ALP	GTTGACACCTGGAAGAGCTT	GTTCCTGTTCAGCTCGTACTG
RUNX2	TGGTTACTGTCATGGCGGGTA	TCTCAGATCGTTGAACCTTGCTA
GAPDH	GGAGCGAGATCCCTCCAAAAT	GGCTGTTGTCATACTTCTCATGG

### Cell proliferation assay

The hDPSCs were seeded in a 96-well plate (Corning, USA) at a density of 2 × 10^3^ cells per well and treated with or without 1 μg/mL LPS for 24 h. The medium was then changed to 50% CGF, 25% iRoot BP Plus extract or their combination, which was defined as CGF-iRoot BP Plus, and replaced with fresh culture medium every 2 days. The proliferation of hDPSCs on days 1, 4, and 7 was evaluated using the Cell Counting Kit-8 assay. The absorbance at a wavelength of 450 nm was determined.

### Cell cycle analysis and apoptosis assay

The hDPSCs were seeded in a 6-well plate (Corning, USA) at a density of 2 × 10^5^ cells per well. The medium was changed to 50% CGF, 25% iRoot BP Plus or CGF-iRoot BP Plus and replaced with fresh culture medium every 2 days. The cell cycle distribution was analysed on day 1, and the populations of apoptotic and nonapoptotic cells were evaluated on days 1, 4, and 7.

For the cell cycle assay, the cells were collected and fixed in 70% ethanol. After washing with PBS, the hDPSCs were dissolved in a hypotonic buffer containing propidium iodide (PI) and collected via flow cytometry. The acquired data were analysed with Cell Quest version 3.1 software (BD Biosciences, USA) according to the manufacturer’s instructions.

For the apoptosis assay, the hDPSCs were suspended in 1 × binding buffer. The suspension was stained with 5 μl of annexin V and PI, gently vortexed and incubated for 15 min at room temperature in the dark. After staining, flow cytometry was performed.

### Real-time quantitative polymerase chain reaction

The hDPSCs were treated with 1 μg/mL LPS for 24 h and divided into four groups, in which the medium was changed to 50% CGF (CGF group), 25% iRoot BP Plus extract (BP group), CGF-iRoot BP Plus, or αMEM (normal control group, NC) for 24 h. The gene expression levels of TNF-α, IL-1β, IL-6, IL-4 and IL-10 (BGI, Shenzhen, China) were determined by real-time quantitative PCR using SYBR Green Mix (Thermo Fisher, Waltham, MA, USA). The primer sequences are listed in Table 1.

In addition, LPS-induced inflammatory hDPSCs from the four groups were collected to evaluate osteo/odontogenic differentiation on day 14. The RNA-Quick Purification Kit (Yishan, Baoshan, Shanghai, China) was used to extract total RNA according to the manufacturer’s instructions. cDNA was then synthesized using the PrimeScriptTM RT Reagent Kit (TaKaRa Co., Kyoto, Japan). Real-time quantitative PCR was performed using Fast SYBR Green Master Mix (Thermo Fisher, Waltham, MA, USA) and gene-specific primers. The messenger RNA expression levels of ALP, runt-related transcription factor 2 (RUNX2), and osteocalcin (OCN) were measured and calculated using the “ΔCt” method. GAPDH was chosen as a housekeeping gene, and the relative expression levels of mRNAs were normalized to that of GAPDH. Primer sequences (BGI, Shenzhen, China) are also shown in Table 1.

### In vivo study

#### Preparation of CGF from rats

CGF from rats was used in the in vivo study. Venous blood (10 mL) was collected from the rats and immediately centrifuged in a centrifuge device as described above to prepare CGF. The CGF membrane was then cut into 1 mm^2^ fragments, soaked in iRoot BP Plus extract for 24 h and defined as CGF-iRoot BP Plus.

#### Scanning electron microscopy and energy-dispersive X-ray analysis

Both CGF and CGF-iRoot BP Plus were fixed in 2.5% glutaraldehyde solution for 3 h and dehydrated serially with ethanol solutions. The ultrastructure was observed at 6000 × magnification, and the elemental composition was analysed by scanning electron microscopy-energy-dispersive X-ray spectrometry (SEM–EDX) (Gemini 500; ZEISS, Oberkochen, Baden-Württemberg, Germany).

#### Vital pulp therapy assay

Thirty eight-week-old male S-D rats weighing 180–250 g were purchased from the Laboratory Animal Center of Sun Yat-sen University, and the left and right maxillary first molars were used for experimental pulpitis (total teeth *N* = 60). The sixty teeth were randomly divided into 5 experimental groups, the LPS group (exposed pulp with LPS treatment, *N* = 12 from 6 rats), CGF group (LPS-exposed pulp with CGF treatment, *N* = 12 from 6 rats), BP group (LPS-exposed pulp with iRoot BP Plus extract treatment, N = 12 from 6 rats) and CGF-iRoot BP Plus group (LPS-exposed pulp with CGF-iRoot BP Plus, *N* = 12 from 6 rats), and the PBS group (exposed pulp with PBS treatment, *N* = 12 from 6 rats) was used as a control. Each group was divided into 3 subgroups according to 3 different time points (1, 7 and 28 days, *N* = 4 from 2 rats at each time point).

Rat pulpitis was induced according to a previous study [29]. After the induction of anaesthesia via intraperitoneal injection of 10% chloral hydrate, the surfaces of the maxillary first molars were disinfected with 5.25% sodium hypochlorite. The pulp of all experimental teeth was mechanically exposed on the occlusal surface using a high-speed handpiece and a round diamond bur (MANI, Japan). Pulp exposure was confirmed with a sterile size 10 K-file and enlarged to a size 30 K-file. Haemostasis was achieved with 5.25% sodium hypochlorite, and 2 μL LPS solution (10 mg/mL) was injected into the pulp. Then, the injured sites were filled with PBS, CGF membrane, iRoot BP Plus extract and CGF-iRoot BP Plus. The site was sealed with light-cured glass ionomer cement (3 M, USA), followed by flowable resin (3 M, USA) restoration.

#### Histologic evaluation

On days 1, 7 and 28 after vital pulp therapy, the animals were sacrificed. The involved teeth and adjacent alveolar bone were isolated and fixed in 4% paraformaldehyde (Servicebio, Wuhan, China) for 24 h at 4 °C. The samples were demineralized in 10% EDTA for 2–3 months at 37 °C and then embedded in paraffin. Sections with a thickness of 4 μm were prepared in the mesiodistal direction for haematoxylin–eosin (HE) staining. Histologic sections were independently evaluated by 2 examiners in a blinded manner. Inflammation was scored according to the Dentistry-Evaluation of Biocompatibility of Medical Devices Used in Dentistry (ISO-7405–2018). Before grading, the interrater reliability test showed that the intraclass correlation coefficient was higher than 0.75 (*P* < 0.05), demonstrating good consistency between the two examiners.

#### Immunohistochemistry

Tissue sections were dewaxed and washed with PBS 3 times. To perform antigen repair, the slides were placed in 0.01 M citrate acid buffer and boiled for 20 min using a microwave oven. After cooling to room temperature, the sections were treated with 3% BSA and 0.3% Triton-100 × in PBS for 1 h. Then, the slides were incubated overnight with an iNOS (1:200, Proteintech, China) or Arg-1 (1:50, Cell Signaling Technology, USA) primary antibody at 4 °C. After washing with PBS, the sections were reacted with species-specific horseradish peroxidase (HRP)-conjugated secondary antibodies (1:10,000, Cell Signaling Technology, USA) for 40 min at room temperature. HRP staining was visualized with the DAB chromogen, and haematoxylin was used to stain nuclei. Images were captured using an Aperio AT2 slide scanner (Leica, Germany).

#### Statistical analysis

Quantitative data are presented as the means ± standard deviations (SDs). All data were analysed using SPSS Statistics 26.0 for Windows. All the data coincided with a normal distribution, and one-way analysis of variance (ANOVA) was used for statistical analysis. Dunnett’s t test was performed for post hoc contrasts to compare multiple experimental groups and control groups. For all analyses, *P* < 0.05 was regarded as statistically significant.

## Results

### hDPSC mesenchymal stem cell characteristics

**Fig. 1:**
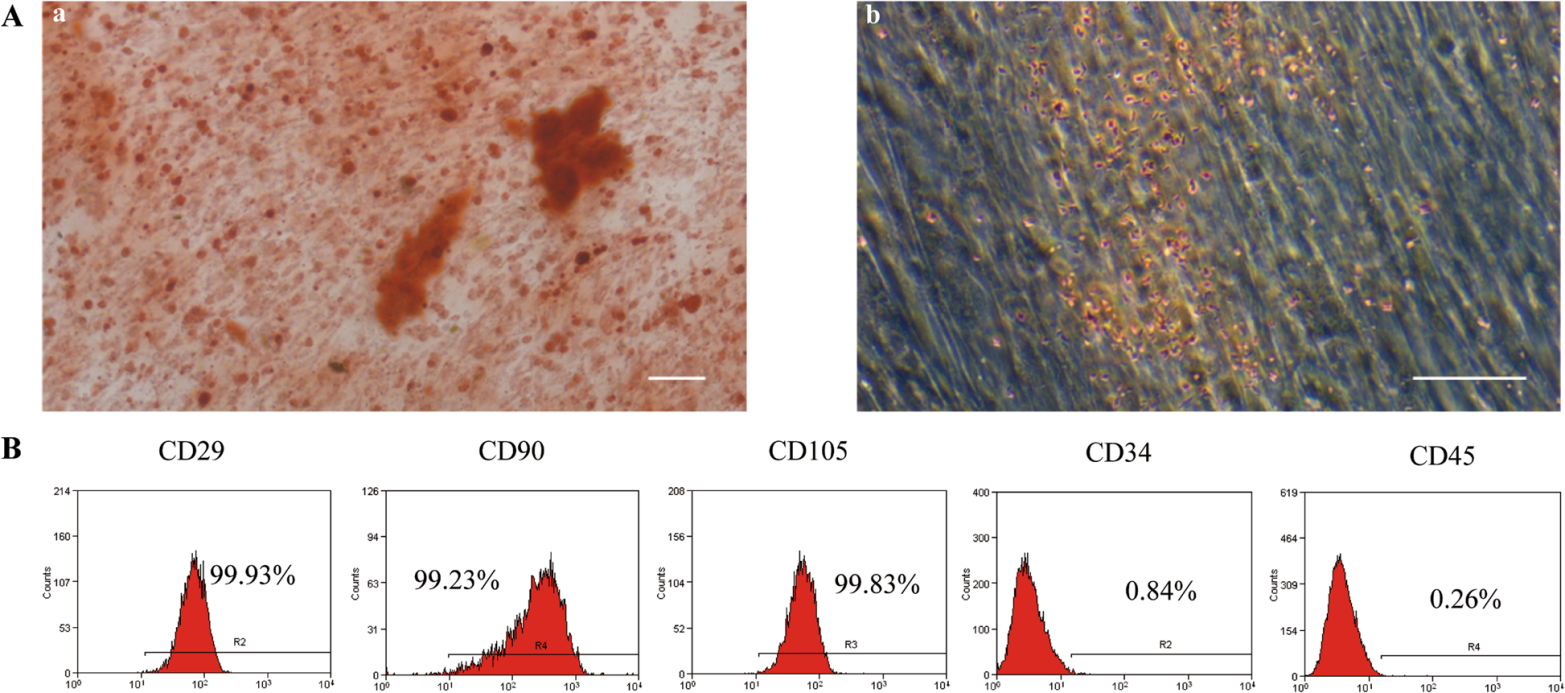
Isolation and identification of hDPSCs. **A** Osteogenesis and lipid induction. a: Representative image of mineralized nodules under a microscope (50x); b: representative image of lipid droplets under a microscope (100x). Scale bar, 100 μm. **B** Representative flow cytometry results showing the expression patterns of CD29, CD90, CD105, CD34 and CD45 in P3 hDPSCs

The primary hDPSCs showed plastic adherence and exhibited a spindle shape. After 3 weeks of osteogenic and adipogenic induction, mineralized nodules and lipid droplets were observed with alizarin red and oil red O staining, respectively (Fig. 1A). Flow cytometry analysis indicated that hDPSCs expressed the mesenchymal stem cell-related antigens CD29, CD90, and CD105 but did not express the haematopoietic cell antigens CD45 and CD34 (Fig. 1B).

### Biocompatibility of CGF and iRoot BP Plus extract in hDPSCs

**Fig. 2:**
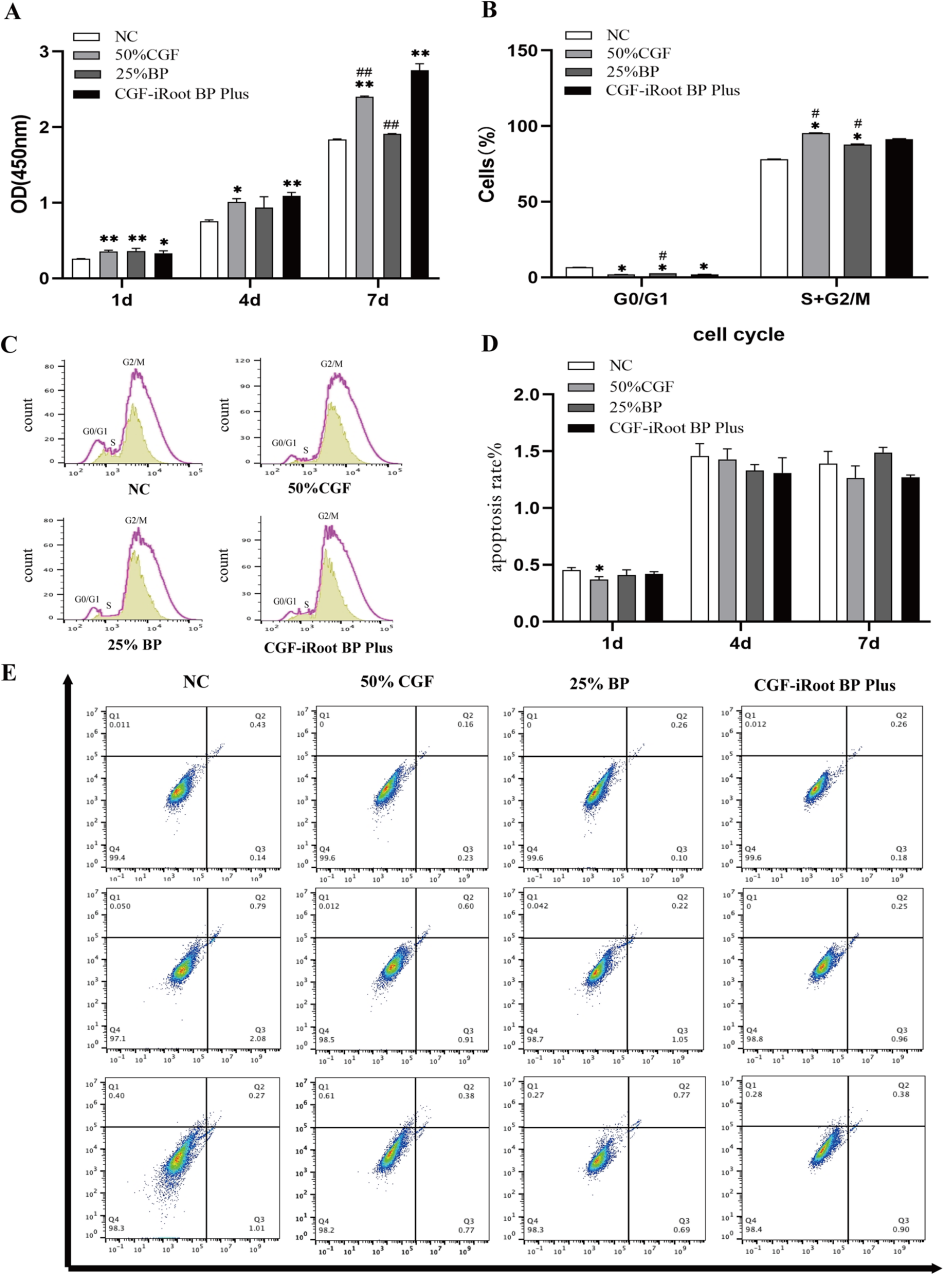
Biocompatibility of CGF and iRoot BP Plus extract in hDPSCs. **A** The proliferation of hDPSCs stimulated by 25% BP, 50% CGF and CGF-iRoot BP Plus. **B** Effects of 50% CGF, 25% BP, and CGF-iRoot BP Plus on the cell cycle of hDPSCs. **C** Flow cytometry to evaluate the cell cycle in hDPSCs after treatment with 50% CGF, 25% BP and CGF-iRoot BP Plus. **D** Effects of 50% CGF, 25% BP and CGF-iRoot BP Plus on the apoptosis rate of hDPSCs. **E** Flow cytometry analysis of the apoptosis rate of hDPSCs after treatment with 50% CGF, 25% BP and CGF-iRoot BP Plus. (*: *P* < 0.05, compared with the NC group, **: *P* < 0.01, compared with the NC group, #: *P* < 0.05, compared with the CGF-iRoot BP Plus group, ##: *P* < 0.01, compared with the CGF-iRoot BP Plus group)

The results of the proliferation assay showed that 50% CGF, 25% iRoot BP Plus extract and CGF-iRoot BP Plus enhanced the proliferation of hDPSCs. Compared with 50% CGF or 25% iRoot BP Plus extract alone, CGF-iRoot BP Plus was significantly more effective in promoting the proliferation of hDPSCs on day 7 (*P* < 0.05) (Fig. 2A).

The cell cycle assay showed that compared with the control group, all the experimental groups had a significantly reduced proportion of cells in G0/G1 phase and an increased proportion of cells in S phase (*P* < 0.05). The CGF-iRoot BP Plus group had more cells in S phase than the 50% CGF group or the 25% iRoot BP Plus extract group (*P* < 0.05) (Fig. 2B, C). In the apoptosis assay, all the experimental groups had no significant effects on the apoptosis of hDPSCs from day 1 to day 7, except for the 50% CGF group on day 1 (Fig. 2D, E).

### The effects of LPS on hDPSC proliferation and inflammatory responses

**Fig. 3:**
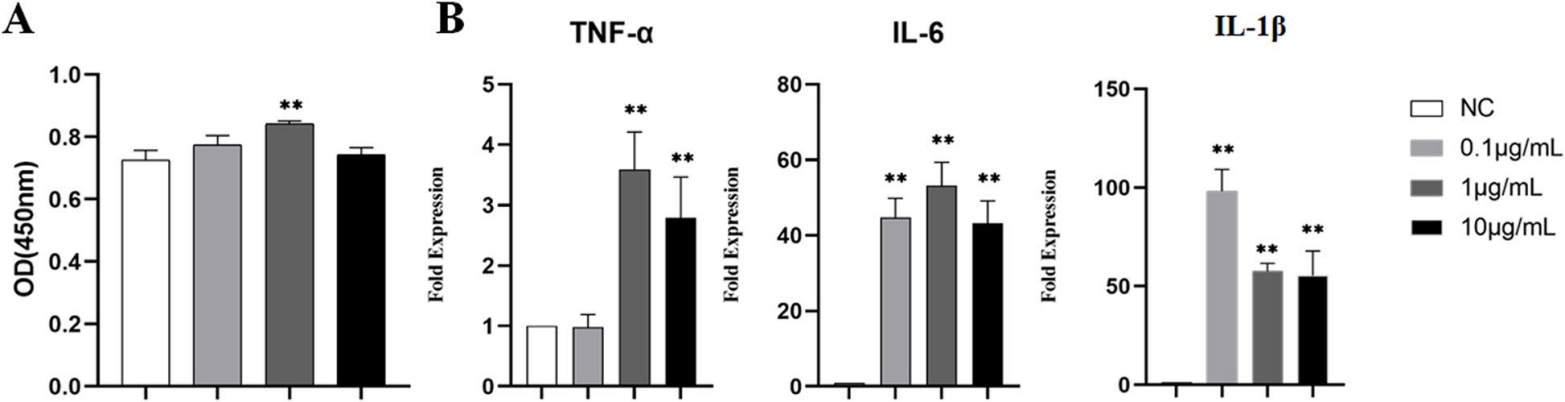
Effects of LPS on cell proliferation and the expression of inflammatory cytokines in hDPSCs after 24 h treatment. **A** Effects of LPS on the proliferation of hDPSCs. **B** Effects of 1 μg/mL LPS stimulation on the expression of inflammatory cytokines in hDPSCs (NC: negative control, **: *P* < 0.01, compared with the negative control group)

After 1 day of LPS (0.1, 1 and 10 μg/mL) treatment, the proliferation of hDPSCs in the 1 μg/mL LPS group was significantly higher than that of hDPSCs in the negative control group (*P* < 0.05), but no significant increase in cell proliferation was observed in the other two groups (Fig. 3A). In addition, the expression levels of IL-6 and IL-1β were significantly increased after treatment with different concentrations of LPS (*P* < 0.01). Only 1 and 10 μg/mL LPS significantly increased the expression of TNF-α (*P* < 0.01) (Fig. 3B).

### Effects of CGF and iRoot BP Plus on proliferation in LPS-stimulated hDPSCs

**Fig. 4:**
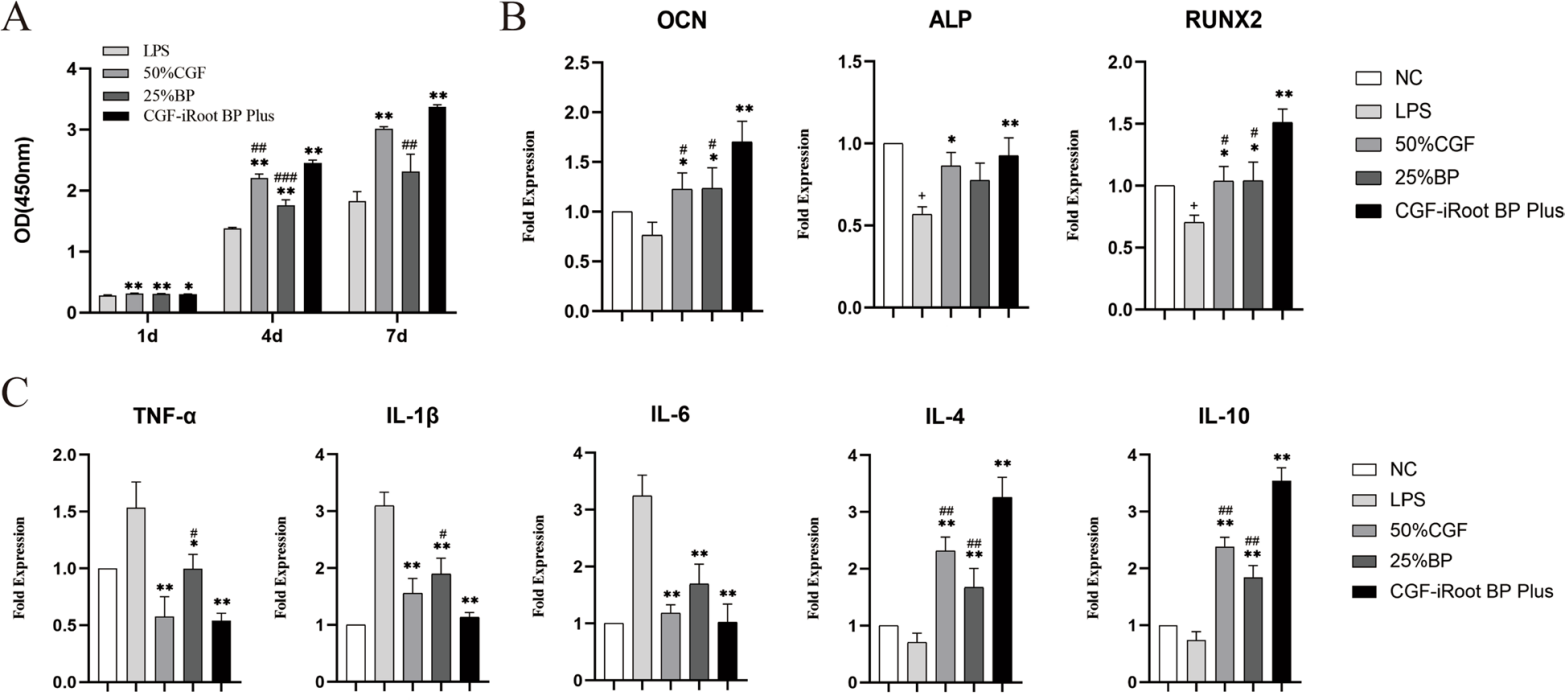
Effects of CGF and iRoot BP Plus on cell proliferation and the expression of inflammatory cytokines and dentinogenesis-related genes in LPS-stimulated hDPSCs. **A** The proliferation of LPS-stimulated hDPSCs at 1, 4, and 7 days. **B** The expression of dentinogenesis-related genes in inflammatory hDPSCs at 14 days. **C** The expression of the proinflammatory factors TNF-α, IL-1β, and IL-6 and the anti-inflammatory factors IL-4 and IL-10 at 24 h (+ : *P* < 0.05, compared with the NC group,*: *P* < 0.05, compared with the LPS group, **: *P* < 0.01, compared with the LPS group, #: *P* < 0.05, compared with CGF-iRoot BP Plus, ##: *P* < 0.01, compared with CGF-iRoot BP Plus, ###: *P* < 0.001, compared with CGF-iRoot BP Plus)

The proliferation of LPS-stimulated hDPSCs after CGF and iRoot BP Plus extract treatment is shown in Fig. 4A. Treatment with 25% iRoot BP Plus extract, 50% CGF and CGF-iRoot BP Plus all promoted the proliferation of inflammatory hDPSCs. Compared with the control treatment, the promotion effects of 50% CGF and CGF-iRoot BP Plus were significantly different on days 1, 4 and 7, while 25% iRoot BP Plus extract significantly increased the proliferation value on days 1 and 4 (*P* < 0.01). In addition, the proliferation rate of the CGF-iRoot BP Plus group was markedly higher than that of the 50% CGF group on day 4, and the difference from the 25% iRoot BP Plus group was significant on days 4 and 7 (*P* < 0.01).

### Transcription of odonto/osteogenic differentiation markers

The mRNA expression levels of OCN, ALP and RUNX2 were decreased after LPS treatment for 24 h, and the levels of ALP and RUNX2 were significantly decreased compared to the control group (*P* < 0.05). However, both 50% CGF and 25% iRoot BP Plus extract rescued the attenuation of osteo/odontogenic differentiation induced by LPS treatment. CGF-iRoot BP Plus significantly upregulated the expression of OCN, ALP and RUNX2 compared to that observed after LPS treatment and led to a significant increase in the expression of OCN and RUNX2 compared to that in the CGF and BP groups (*P* < 0.05) (Fig. 4B).

### Gene expression of inflammatory mediators

The results of real-time quantitative PCR showed that CGF-iRoot BP Plus significantly attenuated the release of TNF-α, IL-1β, and IL-6 in LPS-stimulated hDPSCs on day 1. In addition, the expression levels of IL-4 and IL-10 were upregulated after CGF-iRoot BP Plus treatment in the inflammatory hDPSCs, and the difference was significant in comparison to both 50% CGF and 25% iRoot BP Plus extract (*P* < 0.05) (Fig. 4C).

### Scanning electron microscopy and energy-dispersive X-ray analysis

**Fig. 5:**
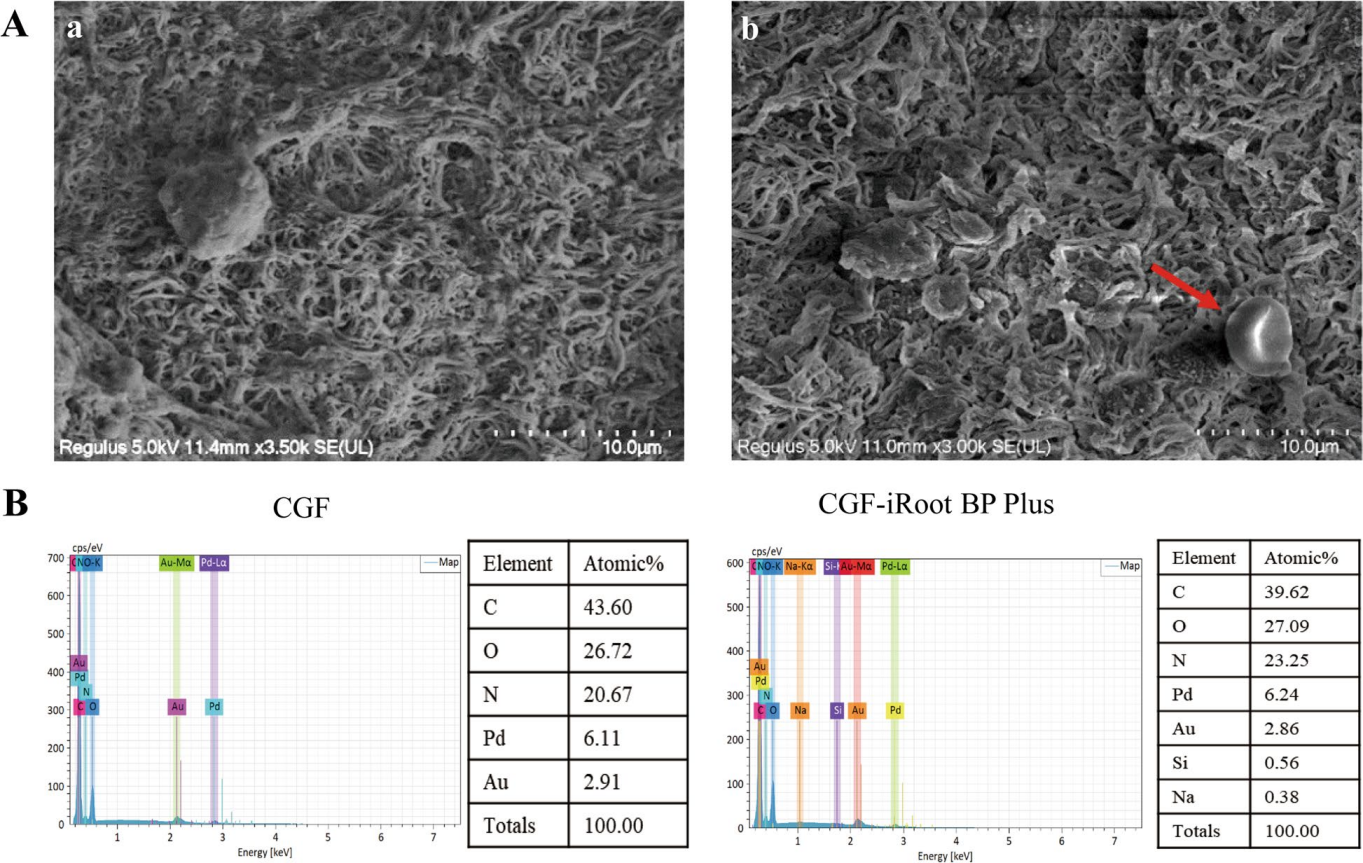
Scanning electron microscopy and energy-dispersive X-ray analysis of CGF and CGF-iRoot BP Plus. **A** Scanning electron microscopy of CGF. a: CGF is composed of a network of interwoven collagen fibres. Scale bar 10 μm; b: CGF is attached to a large number of blood cells (red arrows: red blood cells). Scale bar 10 μm. **B** Energy spectrometry of CGF and CGF-iRoot BP Plus after combination for 1 day

SEM results showed a dense fibrin network on the external surface of rat CGF, with the presence of activated platelets, white blood cells and red blood cells (Fig. 5A).

Energy spectrum analysis showed that rat CGF was mainly composed of the elements C, O and N (Fig. 5B CGF). Si and Na were detected in addition to C, O and N after rat CGF was immersed in iRoot BP Plus extract for 24 h (Fig. 5B CGF-iRoot BP Plus), indicating that CGF could be used as a fibrin scaffold loaded with ions from iRoot BP Plus extract.

### Histologic findings

**Fig. 6:**
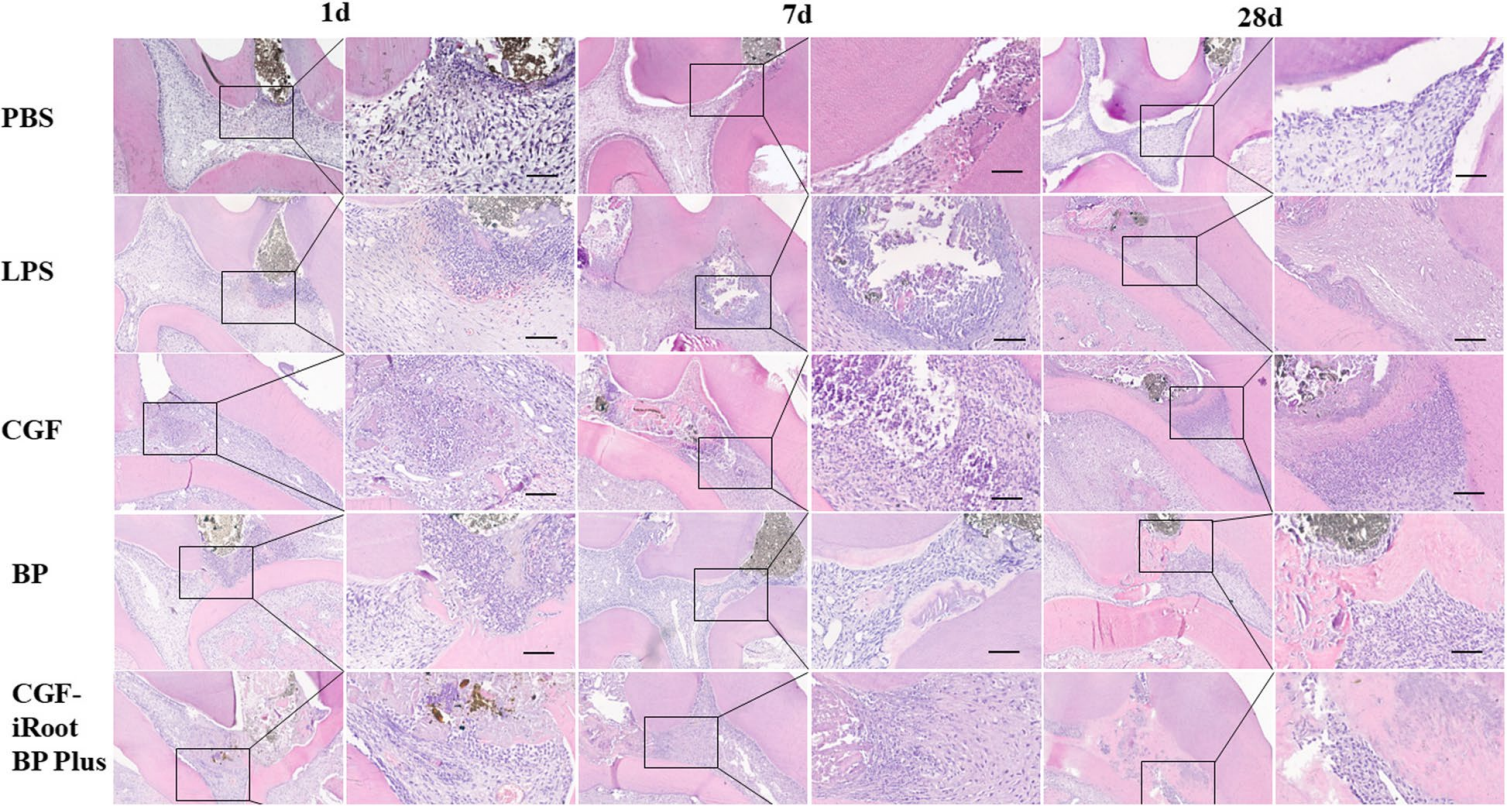
HE staining results from an experimental pulpitis model in rats after direct pulp capping with CGF, iRoot BP Plus and CGF-iRoot BP Plus on days 1, 7 and 28

In the PBS group, a few inflammatory cells and extravasated red blood cells were found beneath the injured site at day 1. Slight edema of the odontoblast layer was also observed around the injured site. On day 7, odontoblast oedema was alleviated, and there was less extravasation of red blood cells. The rest of the pulp had recovered from inflammation and had become normal. On day 28, the pulp tissue below the injured site appeared normal, without inflammatory cells (Fig. 6).

In the LPS group, the teeth presented more severe and rapid signs of pulp inflammation. On day 1, acute inflammatory exudation was observed. The blood vessels were dilated, with red blood cell exudation and tissue oedema. A large number of inflammatory cells accumulated at the injured site and infiltrated towards the coronal pulp. The odontoblast layer was significantly swollen, and the surrounding pulp tissue was disorganized (Fig. 6). On day 7, an increasing number of inflammatory cells accumulated beneath the injured site, and a localised abscess formed with slight necrosis. The edema of the odontoblast layer was relieved. On day 28, the coronal and upper-middle pulp tissue became extensively necrotic, and numerous inflammatory cells infiltrated towards the apical pulp.

In the CGF group, there was dense infiltration of inflammatory cells beneath the exposed pulp on day 1. Slightly dilated blood vessels and red blood cell exudation were observed. On day 7, inflammatory cells diffusely infiltrated towards the pulp chamber, and the morphologic condition of the apical pulp was normal, without any inflammatory cells. On day 28, calcified tissue was generated at the injured site, which appeared structurally looser than the normal dentin. The cells below the newly formed tissue were squeezed, with a slightly disordered organization and morphology, whereas the structure of the apical pulp tissue was normal and characterized by the absence of inflammatory cells and well-organized pulp tissues (Fig. 6).

In the BP group, inflammatory cells accumulated beneath the injured site, and the surrounding odontoblasts showed slight oedema on day 1. On day 7, disordered pulp tissue with inflammatory cell infiltration was observed in close proximity to the injured site. A small amount of reparative dentin was formed along the wall of the pulp chamber but did not seal the injured site. On day 28, a calcified dentin bridge was deposited beneath the injured site to seal the wound surface, in which cell debris was scattered. In addition, the cells below the dentin bridge showed palisading alignment. The apical pulp tissue appeared normal, without obvious inflammatory cell infiltration (Fig. 6).

In the CGF-iRoot BP Plus group, a small number of inflammatory cells and red blood cell exudation were observed in the vicinity of the injured site on day 1. The adjacent pulp tissue presented slight edema. Remission of the inflammatory response occurred on day 7 and was accompanied by better-organized pulp tissues than those observed on day 1. On day 28, a mass of hard tissue deposition was observed underneath the injured site. The thick dentin bridge appeared continuous and even sealed the injured site. The apical pulp tissue appeared normal, without obvious inflammatory cell infiltration (Fig. 6).

**Fig. 7:**
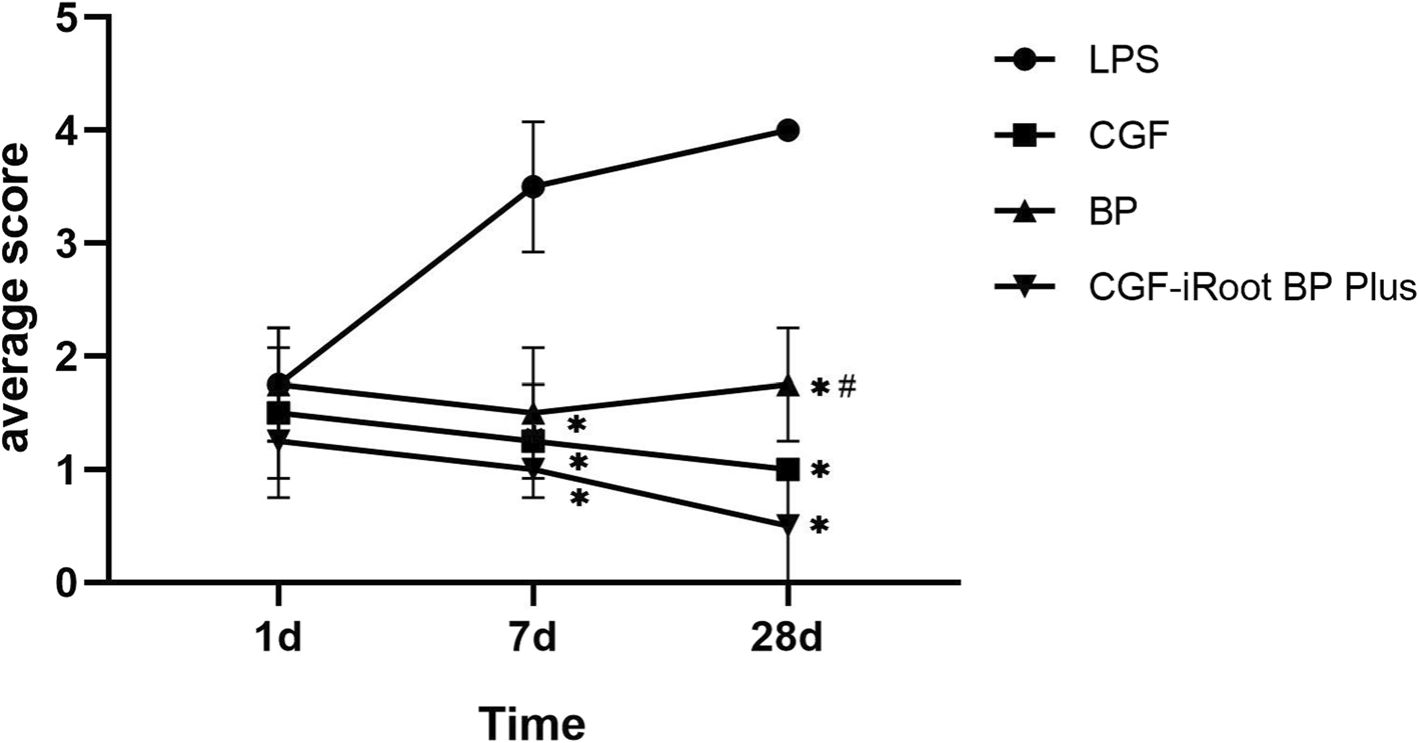
Serial changes in inflammation scores (*: *P* < 0.05 compared to the LPS group, # *P* < 0.05, compared with the CGF-iRoot BP Plus group)

The distributions of inflammatory scores are shown in Table 2 and Fig. 7. After LPS treatment, the inflammatory score obviously rose from day 1 to day 28. The application of CGF, BP and CGF-iRoot BP Plus significantly decreased the inflammatory scores in comparison with those of the LPS group on day 7 and day 28 (*P* < 0.05). The average score of the BP group was higher than that of the CGF and CGF-iRoot BP Plus groups from day 1 to day 28 and showed a marked difference from that of the CGF-iRoot BP Plus group on day 28 (*P* < 0.05). From day 7 to day 28, the score of the BP group increased, while the score of the CGF group remained the same as that on day 7. The score of the CGF-iRoot BP Plus group continued to decrease.

**Table 2:** Grading of inflammation in rat pulp tissue

Group	1 d	7 d	28 d
PBS	0	0	0
LPS	1.75	3.50	4.00
CGF	1.50	1.00*	1.00*
BP	1.75	1.50*	1.75*^,^^**#**^
CGF-iRoot BP Plus	1.25	1.00*	0.50*

^*^: *P* < 0.05, compared with the LPS group

^#^ *P* < 0.05, compared with the CGF-iRoot BP Plus group

### Immunohistochemical staining of M1 and M2 macrophages

**Fig. 8:**
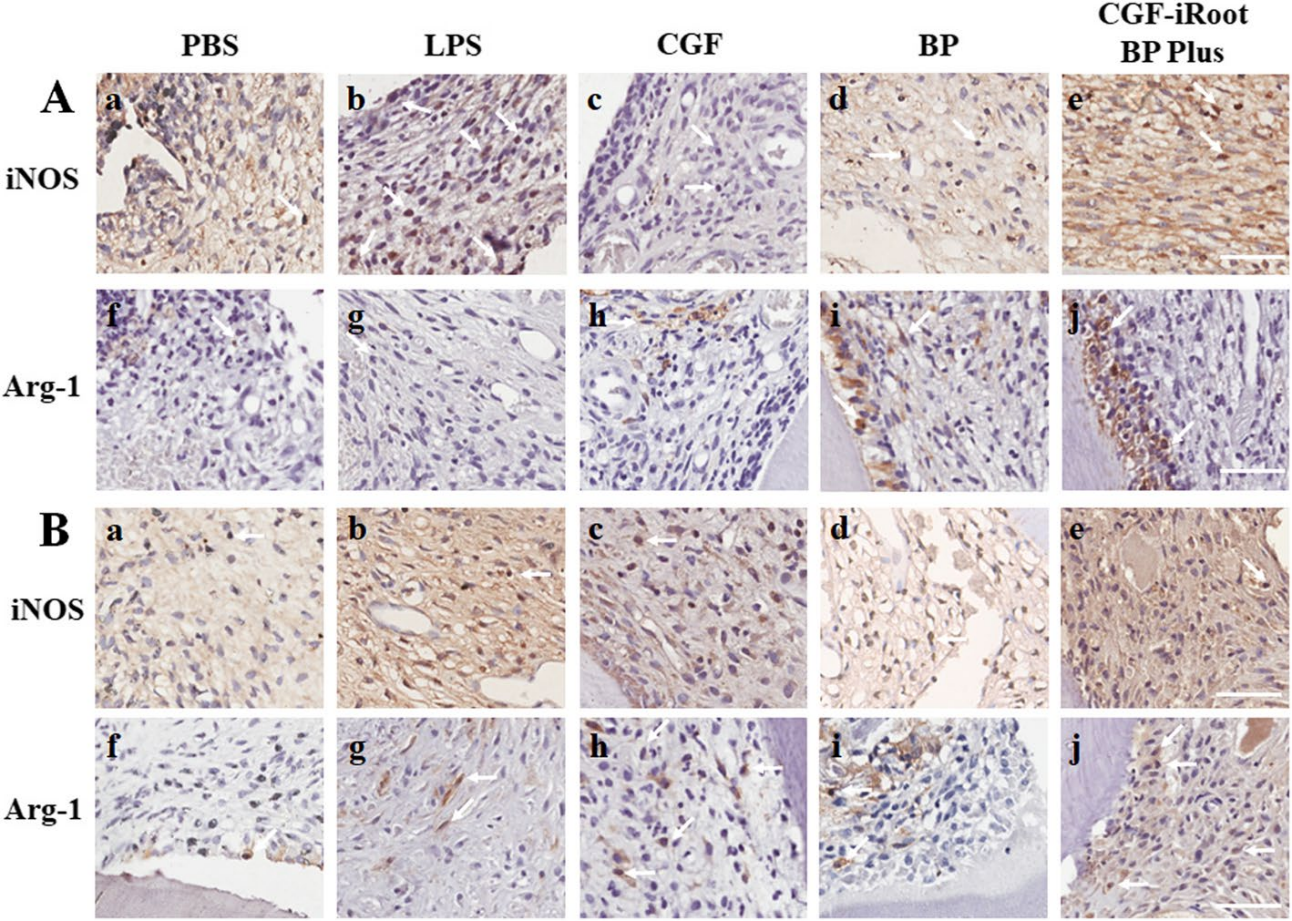
Immunohistochemical staining of iNOS and Arg-1 in experimentally induced pulpitis. **A**. Expression of iNOS and Arg-1 on day 1 postinjury. a-e: Infiltration of iNOS + cells in dental pulp; f-j: scattered Arg + cells in dental pulp. **B**. Expression of iNOS and Arg-1 on day 7 postinjury. a-e: Distribution of iNOS + cells in dental pulp; f-j: infiltration of Arg + cells in dental pulp. Arrows indicate iNOS + /Arg-1 + cells. Scale bar: 50 μm

Immunohistochemical staining demonstrated the presence of M1/M2-like macrophages during the dental pulp healing process (Fig. 8). iNOS and Arg-1 antibodies resulted in brownish-yellow, patchy staining in the cytoplasm, and the stained cells were identified as M1- or M2-like macrophages, respectively. On day 1, PBS caused a mild immune response, as few iNOS + or Arg-1 + cells were found in the pulp tissue. Compared with the BP, CGF and CGF-iRoot BP Plus groups, the LPS group showed the largest number of iNOS + cells around the injured site. In addition, a few Arg-1 + cells were found at the exposed site in all groups treated with LPS. Interestingly, the BP and CGF-iRoot BP Plus groups exhibited sheet-like staining in the odontoblast layer. On day 7, in each group, the number of iNOS + cells was decreased, while the number of Arg-1 + cells was increased compared with that on day 1. Scattered Arg-1 + cells were found in the LPS group, and the CGF and CGF-iRoot BP Plus groups had more Arg-1 + cells than the LPS and BP groups.

## Discussion

CGF is the third generation of platelet concentrates, and it contains more growth factors and a harder fibrin structure than previous platelet concentrates [30, 31], exhibiting superior regenerative capacity and biomaterial potential [32–34]. During dentin/pulp regeneration, various growth factors work synergistically rather than individually [35]. Thus, the presence of multiple growth factors in CGF may be of greater importance than a single bioactive protein. Several studies have elucidated the potential of CGF as a pulp capping material for VPT [31, 36, 37]. However, the lack of temporal degradation control and the limitation of inadequate mechanical strength to support coronal restoration further restrict the application of CGF [38, 39]. In the present study, the ultrastructure of CGF was confirmed as a dense fibrin network with porous structures, which was in good agreement with a previous report [31].

As a novel calcium silicate–based nanoparticulate bioceramic putty, iRoot BP Plus exhibits excellent sealing properties, comparable or superior biocompatibility, osteoconductive potential [40–42] and significantly less discolouration than the traditional calcium silicate cement MTA [43]. When CGF was immersed in iRoot BP Plus extract, the ions released from iRoot BP Plus, such as Si and Na, were detected in CGF, indicating that CGF combined with iRoot BP Plus may synergistically act as a biological scaffold in which dental pulp cells can be embedded for functional effects. To further demonstrate the biological effect of the composite material on hDPSCs, 50% CGF and 25% iRoot BP Plus extract were selected for the in vitro study based on our pilot study.

Pulp capping materials directly contact vital pulp tissue, and suitable biological properties are one of the most clinically relevant factors. Previous studies have reported negligible cytotoxicity of CGF and iRoot BP Plus to various cells in vitro [31, 37, 44]. In the present study, the results further revealed that CGF combined with iRoot BP Plus significantly increased the proportion of cells in S stage and did not induce apoptosis in hDPSCs, suggesting that the combination of these two materials may preserve cell viability and have the potential to promote self-renewal of dental pulp.

The hypothesis that reparative processes occur in the dental pulp only when infection and inflammation are under control is generally accepted [45]. Thus, it is crucial to control inflammation with pulp capping materials to achieve odontogenic differentiation of hDPSCs and the formation of dentin bridges via VPT [46]. TNF-α, IL-1β, and IL-6 are well-known proinflammatory cytokines and can be elicited by LPS in hDPSCs [22, 47]. In the present study, LPS was used to stimulate an inflammatory microenvironment during pulpitis. As expected, LPS treatment significantly increased the levels of TNF-α, IL-1β, and IL-6 mRNA in hDPSCs. However, the expression of these cytokines was decreased, while the expression of the anti-inflammatory factors IL-4 and IL-10 was increased by treatment with CGF, iRoot BP Plus and their combination. Moreover, the above changes in the combination group were significantly different from those in the iRoot BP Plus group, implying that the combination of CGF and iRoot BP Plus may regulate the inflammatory response of injured pulp.

A rat model of experimental pulpitis was established as previously reported [29] and subjected to direct pulp capping. The inflammatory scores of the rat pulp in the early stage indicated the anti-inflammatory effects of iRoot BP Plus and CGF, which may be partly attributed to the antibacterial activity of both materials [48, 49]. Furthermore, iRoot BP Plus may have a favourable effect on the crosstalk between MSCs and macrophages and convert DPSCs to an anti-inflammatory and prorepair phenotype [50]. The IHC results after staining for iNOS and Arg-1 suggested that CGF may be capable of regulating macrophage polarization of hDPSCs to the M2 phenotype, which was similar to a recent report demonstrating that CGF extract promoted THP-1 macrophage polarization towards the M2 phenotype, with upregulated CD163 expression [33]. Notably, our results also showed that the inflammation score tended to increase in the iRoot BP Plus group in the late stage, suggesting the limited anti-inflammatory effect of the single pulp capping material. In addition, the inflammation score was sustained in the CGF group and continued to decrease in the combination group, which may be due to the slow release of various growth factors in CGF [51]. Comprehensively, CGF and iRoot BP Plus may have a synergistic anti-inflammatory effect on injured pulp.

Cell proliferation and odonto/osteogenic differentiation of the incorporated cells are critical steps for pulp regeneration and dentin formation. Human dental pulp stem cells isolated from inflamed pulp (I-DPSCs) derived from carious teeth with symptomatic irreversible pulpitis were reported to have stemness and multidifferentiation potential [52], highlighting the possibility of successful VPT for inflamed pulp. Previous studies have confirmed the ability of CGF and iRoot BP Plus to promote the proliferation and differentiation of various odontogenic cells [37, 53]. In our study, CGF combined with iRoot BP Plus extract was verified to promote the proliferation of hDPSCs whether under LPS stimulation or not. Although CGF or iRoot BP Plus extract alone was found to promote the proliferation of hDPSCs, the effect of their combination on LPS-stimulated hDPSCs was significantly stronger, implying that these two materials can work synergistically and accelerate the proliferation of hDPSCs under LPS-stimulated conditions.

The odonto/osteogenic differentiation of hDPSCs may be identified by the expression of several genes, including ALP, Runx2, and OCN [22]. As shown in the present study, LPS treatment suppressed the expression of the odontoblastic-related genes ALP, Runx2, and OCN in hDPSCs, which was consistent with a previous report showing that LPS inhibited the upregulation of RUNX2 and reduced the expression of odontoblast-associated proteins [54]. Cotreatment with LPS and CGF has been revealed to increase the mRNA expression of Runx2 and OCN compared with LPS treatment alone [22]. Consistently, the present study demonstrated that the gene expression levels of ALP, Runx2, and OCN were upregulated by CGF and iRoot BP Plus, especially when both materials were combined under LPS stimulation. These results demonstrated that the detrimental effect on the odontogenic differentiation capability of hDPSCs caused by LPS may be rescued by the combination of CGF and iRoot BP Plus. The in vivo study further clarified that a continuous dentin bridge was formed to seal the injury site on the 28th day after the application of CGF combined with iRoot BP Plus. Interestingly, on the 7th day, a small amount of reparative dentin was observed in the iRoot BP Plus group but not in the CGF group or the combination group. On the 28th day, all three experimental groups showed calcification underneath the injury site. We speculated that this may be due to the different mechanisms of odonto/osteogenic differentiation in hDPSCs treated with CGF and iRoot BP Plus. The osteogenic/odontogenic potential of iRoot BP Plus has been attributed to Ca and Si ion release [55–57], and the specific release kinetics of a variety of growth factors from CGF, including PDGF-BB, TGF-β1, BMP-2 and VEGF, may account for the mineralization of hDPSCs. These growth factors were released slowly and reached their maximum on day 14 (VEGF) or day 21 (TGF-β1 and BMP-2) after CGF preparation. TGF-β1 and BMP-2 levels may remain high for up to 28 days [51].

Overall, we confirmed that compared with CGF or iRoot BP Plus alone, the combination of CGF and iRoot BP Plus can regulate the inflammatory response and facilitate hard tissue formation in inflamed pulp, inducing synergistic effects on pulp cell activity. Further research exploring the response of stem cells in vivo and the mechanisms of this synergistic effect will shed light on the application of this composite material for dental pulp repair.

## Conclusion

The present study revealed that the combination of CGF and iRoot BP Plus promoted the proliferation and differentiation of hDPSCs under LPS-stimulated conditions in vitro and exhibited potential for anti-inflammatory effects and pulp healing in vivo. A synergistic effect of the combination treatment was observed compared to CGF or iRoot BP Plus alone. Therefore, the combination of CGF and iRoot BP Plus may be a promising pulp capping biomaterial in unknown inflammatory conditions, revealing this DPC agent as an additional treatment option for pulpitis.

## Data Availability

The datasets analysed during the current study are available from the corresponding author on reasonable request.

## Abbreviations

CGF: Concentrated growth factor
hDPSCs: Human dental pulp stem cells
VPT: Vital pulp therapy
ESE: European society of endodontology
AAE: American association of endodontists
MTA: Mineral trioxide aggregate
DPC: Direct pulp capping
PDGF-BB: Platelet-derived growth factor-BB
TGF-β1: Transforming growth factor β-1
IGF-1: Insulin-like growth factor-1
VEGF: Vascular endothelial growth factor
bFGF: Basic fibroblast growth factor
DSPP: Dentin sialophosphoprotein
DMP-1: Dentin matrix protein-1
ALP: Alkaline phosphatase
αMEM: α-Minimum essential medium
RUNX2: Runt-related transcription factor 2
OCN: Osteocalcin
